# Differential gene expression in response to fungal pathogen exposure in the aquatic invertebrate, *Daphnia dentifera*

**DOI:** 10.1002/ece3.10354

**Published:** 2023-07-28

**Authors:** Emily E. Terrill Sondag, Tara E. Stewart Merrill, Jenny Drnevich, Jessica R. Holmes, Eva K. Fischer, Carla E. Cáceres, Lynette R. Strickland

**Affiliations:** ^1^ Department of Evolution, Ecology, and Behavior, School of Integrative Biology University of Illinois Urbana‐Champaign Urbana Illinois USA; ^2^ Coastal and Marine Laboratory Florida State University St. Teresa Florida USA; ^3^ High Performance Computing in Biology University of Illinois Urbana‐Champaign Urbana Illinois USA; ^4^ Department of Biology Boston University Boston Massachusetts USA

**Keywords:** *Daphnia*, invertebrate immunity, *Metschnikowia*, pathogen, RNA‐sequencing

## Abstract

While vertebrate immune systems are appreciated for their complexity and adaptability, invertebrate immunity is often considered to be less complex. However, immune responses in many invertebrates likely involve sophisticated processes. Interactions between the crustacean host *Daphnia dentifera* and its fungal pathogen *Metschnikowia bicuspidata* provide an excellent model for exploring the mechanisms underlying crustacean immunity. To explore the genomic basis of immunity in *Daphnia*, we used RNA‐sequencing technology to quantify differential gene expression between individuals of a single host genotype exposed or unexposed to *M. bicuspidata* over 24 h. Transcriptomic analyses showed that the number of differentially expressed genes between the control (unexposed) and experimental (exposed) groups increased over time. Gene ontology enrichment analysis revealed that differentially expressed genes were enriched for immune‐related molecules and processes, such as cuticle development, prostaglandin, and defense response processes. Our findings provide a suite of immunologically relevant genes and suggest the presence of a rapidly upregulated immune response involving the cuticle in *Daphnia*. Studies involving gene expression responses to pathogen exposure shine a light on the processes occurring during the course of infection. By leveraging knowledge on the genetic basis for immunity, immune mechanisms can be more thoroughly understood to refine our understanding of disease spread within invertebrate populations.

## Graphical abstract

Invertebrate immunity is often oversimplified despite the likeliness that complex immune mechanisms exist in this group. We leverage a model host‐pathogen system Daphnia dentifera and Metschnikowia bicuspidata to explore the genetic underpinnings of invertebrate immunity. We found that Daphnia have a very dynamic and rapidly upregulated immune response during the first 24 h following pathogen exposure and lay important groundwork for future invertebrate immunological studies by providing a suite of putative immune‐related transcripts and their current Gene Ontology annotations.

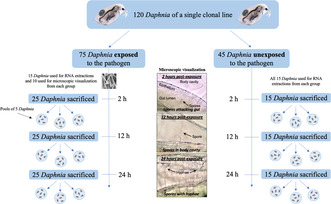

## 1 INTRODUCTION

Vertebrates have traditionally been the focus of immunological studies, however, the diversity among invertebrate immune systems paired with their attractiveness as laboratory subjects has resulted in a recent expansion of invertebrate immunological research (Kurtz & Armitage, 2006; Schulenburg et al., 2007). This expansion has revealed similarities in immune processes across vertebrates, invertebrates, and plants, but has also highlighted vast differences among species, most of which are not well understood (Litman & Cooper, 2007). Moreover, vertebrate immune systems are often contrasted with invertebrate immune systems, which obscures the magnitude of potential immunological variation among invertebrate species that represent dozens of animal phyla. As a result of the invertebrate radiation during the Cambrian explosion (520–530 million), the evolutionary trajectories of invertebrates, including diverse parasite taxa, are independent and have been influenced by a variety of environmental challenges (Loker et al., 2004). This long evolutionary history of invertebrates has likely produced unique approaches to resisting parasites immunologically, which can make generalization across phyla difficult (Medzhitov & Janeway, 1997). In short, invertebrates are highly diverse and understudied in terms of their immunological strategies.

The two well‐accepted classes of the immune response are innate immunity, consisting of nonspecific, hard‐wired defense mechanisms, and adaptive or acquired immunity, which is categorized as responses that create and use immunological memory (Litman et al., 2010). Vertebrates have been hallmarked by their possession of an advanced, adaptive immune system (Boehm, 2012). Conversely, invertebrates are typically stereotyped as being immunologically simple and relying solely on an innate immune system (Auld et al., 2010). However, the line that separates innate and acquired immunity has been blurred by recent immunological advancements in multiple phyla (Černý & Stříž, 2019; Kurtz & Franz, 2003; Medzhitov & Janeway, 1997). For example, the squid, *Euprymna scolopes*, maintains a complex mutualistic relationship with the bioluminescent bacterium *Vibrio fischeri*, which resides in the squid's light organ (Visick et al., 2000). The squid avoids the bacterium overrunning its body by keeping it relegated to one area in which the bacterium is beneficial, suggesting that a complex, dynamic immune system is present, involving a strong capacity to differentiate between beneficial and pathogenic bacteria. A second example is the advanced immune role accomplished by the *Down syndrome cell adhesion molecule (Dscam)* via alternative splicing, with homologs present across arthropod species (Armitage et al., 2015; Brites et al., 2013). Alternative splicing of *Dscam* has recently been described to potentially produce 18,000 extracellular receptor isoforms in the fat body and hemocytes, which are involved in pathogen recognition and specificity (Watson et al., 2005). This is similar to the adaptive machinery housed in vertebrates in which immunoglobulins and T receptor cells are generated via recombination of the variable, diversity, and joining gene segments (V(D)J) (Litman et al., 2010). Invertebrates live in a world occupied by diverse, abundant, and rapidly evolving pathogens, including those that castrate or kill their hosts to facilitate transmission (Ebert et al., 2004). To deal with these threats, invertebrates likely possess complex immune responses that have not yet been revealed (Little et al., 2005).

To build on more than a decade of work in the *Daphnia dentifera‐Metschnikowia bicuspidata* system, we move beyond a handful of candidate genes to conduct the first transcriptomic analysis of this system. *Daphnia* are of substantial ecological importance to freshwater ecosystems in the Midwestern United States and serve as an exceptional model system for understanding invertebrate immunity because they lend themselves well to manipulative lab work, observational fieldwork, and genomic techniques (Ebert, 2008; Miner et al., 2012; Taylor et al., 1996; Taylor & Hebert, 1992). *Metschnikowia bicuspidata*, a parasitic ascomycetous yeast, spreads horizontally via needle‐like spores among *Daphnia* individuals (Mendonça‐Hagler et al., 1993; Stewart Merrill & Cáceres, 2018). Pathogens like *M. bicuspidata* have long influenced ecological and evolutionary processes among their hosts, making host resistance vital in maximizing individual fitness when confronted with disease outbreaks among populations (Baucom & de Roode, 2011; Mydlarz et al., 2006). The within‐host life cycle of the *Metschnikowia* fungal pathogen was recently described, revealing five morphologically distinct stages of infection (Stewart Merrill & Cáceres, 2018). In brief, ingested fungal spores penetrate the *Daphnia* host's gut and enter the body cavity, where the spores then develop sequentially into hyphae, sporocysts, conidia, and asci. Each of these stages of infection can be limited by the immune response, so each stage has the potential to produce individual variations in defense mechanisms (Stewart Merrill et al., 2019). This variation likely has a genetic basis (Hall et al., 2010; Hall & Ebert, 2012; Rogalski et al., 2021; Strauss et al., 2015) and could arise through novel genes that are not yet annotated. While many conserved genes play an important role in invertebrate immunity, novel genes may pave the way for adaptive responses and individual variation. Despite a consensus on the probable importance of genetic variation in the immune response, these mechanisms governing *Daphnia* immunity remain mostly unknown (Cáceres et al., 2014).

Research on related host species provides some insight into how *Daphnia* immune responses may operate. The genome of *Daphnia pulex*, a species distantly related to *D. dentifera*, was sequenced, and found to contain homologs of immune‐related genes such as the TOLL pathway present in insects (McTaggart et al., 2009). Such comparative sequencing allows for the detection of known immune‐related genes but does not allow the detection of novel immune mechanisms (Decaestecker et al., 2011). RNA‐sequencing (RNA‐seq), on the contrary, is a deep‐sequencing approach to transcriptomic analysis that provides functional evidence for annotated genes and allows for the detection of transcripts without an existing genome (Geraci et al., 2020; Wang et al., 2009). This enables the detection of the up/down‐regulation of both annotated and unannotated genes, providing insight into non‐homologous, novel immune‐related genes. Decaestecker et al. (2011) analyzed the dynamics of gene expression in *Pasteuria ramosa*‐infected and uninfected *D. magna* by targeting a suite of five putative immune‐related genes and found that there were no significant changes in gene expression among these genes that were expected to be upregulated in response to infection. This suggests that there may be other genes, not yet discovered, governing *Daphnia* immunity. McTaggart et al. (2015) took an RNA‐seq approach to explore transcriptional changes of *D. magna* in response to exposure to a bacterial pathogen (*Pasteuria ramosa*) and showed that the host genome responds rapidly and dynamically to pathogen exposure. Their study yielded several putative immune‐related genes. Shortly after, Lu et al. (2018) investigated the transcriptional response of *D. galeata* in response to exposure to the pathogen *Caullerya mesnili* and found that genes related to immune function and metabolism were downregulated 48 h following parasite exposure. In just these two different host–parasite systems, McTaggart et al. (2015) and Lu et al. (2018) found different transcriptomic responses to infection, which is motivation to cast a broader net and explore immune dynamics in additional host–parasite systems.

Motivated by these discoveries in other *Daphnia* species, we began the search for genes involved in *Daphnia dentifera* immunity. We utilized RNA‐seq to analyze differential gene expression in pathogen‐exposed and unexposed individuals at multiple time points over the first 24 h of infection, which is a critical period for host immune defenses (Rogalski et al., 2021). We expected to find immunologically important genes upregulated in exposed individuals compared to unexposed individuals. We predicted that the suite of differentially expressed genes might contain immune‐related genes previously discovered in *D. magna* or *D. pulex*, such as prophenoloxidase or the TOLL pathway (McTaggart et al., 2009; Mucklow & Ebert, 2003), but also novel, immune‐related genes. We also predicted that we would find a unique response to pathogen exposure in our host–parasite system as compared to previous work (Lu et al., 2018; McTaggart et al., 2015) due to taxonomic differences in the host and parasite and the fundamentally different infection strategies of the pathogens. That is, while *Pasteuria* binds to host cells during early infection, *Metschnikowia* punctures the epithelium, and these fundamentally different infection strategies likely induce different host responses. By taking a broad RNA‐sequencing approach, we characterize the expression patterns of both known and novel immune‐related genes in response to fungal pathogen exposure in *Daphnia*.

## 2 METHODS

### 2.1 Pathogen exposure and sample collection

**FIGURE 1:**
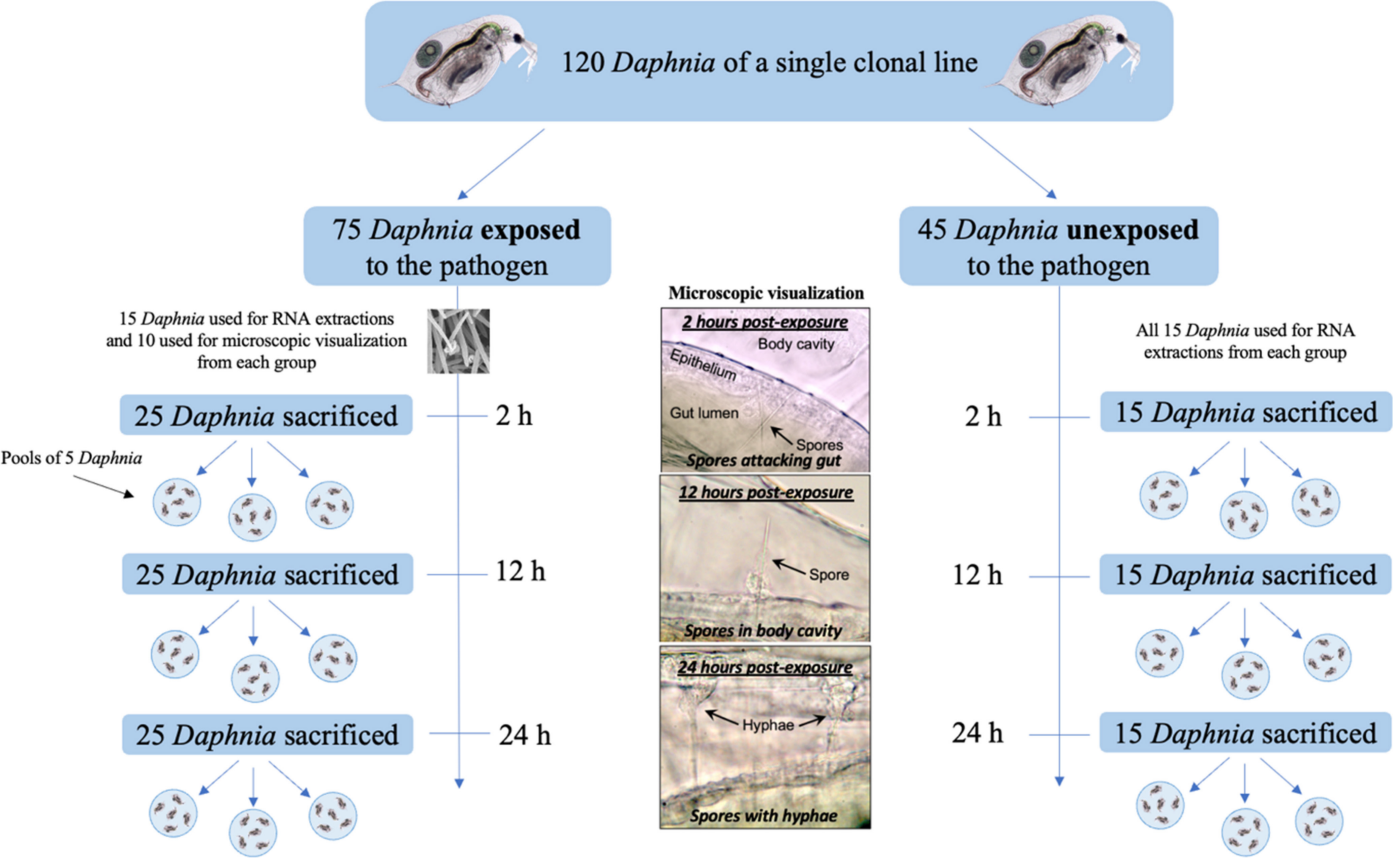
Our experimental design included 120 *Daphnia* of a single clonal line. Individuals were randomly assigned to one of the six factorial combinations of exposure (exposed or unexposed) and time point (2, 12, or 24 h). Fifteen *Daphnia* from each of the six treatments were sacrificed for RNA sequencing, and 10 *Daphnia* from each of the 3 exposed time points were collected for microscopic evaluation to document the progress of infection. Each treatment group of 15 individuals that were sacrificed for RNA sequencing was pooled into three groups of five individuals to ensure sufficient amounts of RNA.

We used a laboratory experiment to quantify differential gene expression in the 24 h following exposure to a pathogen and to align those differences with the development of the pathogen inside the host. To create replicates of our host‐pathogen interaction, we reared individuals of a single *Daphnia dentifera* genotype and a single *Metschnikowia bicuspidata* strain collected in 2003 from lakes in Barry and Kalamazoo County, MI. Prior to the start of the experiment, *Daphnia* were raised for three generations at low densities to standardize maternal effects (Lynch & Walsh, 1998), and *Metschnikowia* were cultured in live hosts of the same *Daphnia* genotype. To begin the experiment, a cohort of same‐aged (<24 h) host neonates were transferred to individual 50 mL falcon tubes containing 45 mL of filtered lake water that was kept at 21°C. *Daphnia* were fed daily with 1 mg C/L of the green algae *Ankistrodesmus falcatus*. On day 8 (when the *Daphnia* had achieved maturity), each *Daphnia* was transferred into an “inoculation chamber”, a 15 mL falcon tube containing 10 mL of filtered lake water. The individuals were then randomly assigned to one of two exposure groups (pathogen‐exposed or unexposed (control)) and one of three time points (2, 12, or 24 h) in a fully factorial design of six treatments (Figure 1). The pathogen‐exposed group received an inoculation of 500 spores/mL of the fungal pathogen *Metschnikowia bicuspidata*. This level of spore concentration ensures that enough spores will be consumed by the host to confidently conclude exposure to the pathogen (Stewart Merrill et al., 2019). The exposure period lasted up to 24 h. During that time, each tube was inverted every 15 min for the first hour, and then hourly for the next 11 h to maintain suspension of *Metschnikowia* spores.

At 2, 12, and 24 h after exposure, 15 *Daphnia* from each of the six treatments were sacrificed, placed into RNAlater, and stored at −20°C and 10 *Daphnia* from each exposure treatment were collected for microscopic evaluation (Total *N* = 120). We split each set of 15 *Daphnia* into three groups of five individuals to ensure a sufficient amount of RNA for extraction and subsequent analysis. This resulted in three pools, or replicates, per treatment for 18 samples total. All individuals used in the experiment were of the same clonal line of *Daphnia*.

**FIGURE 2:**
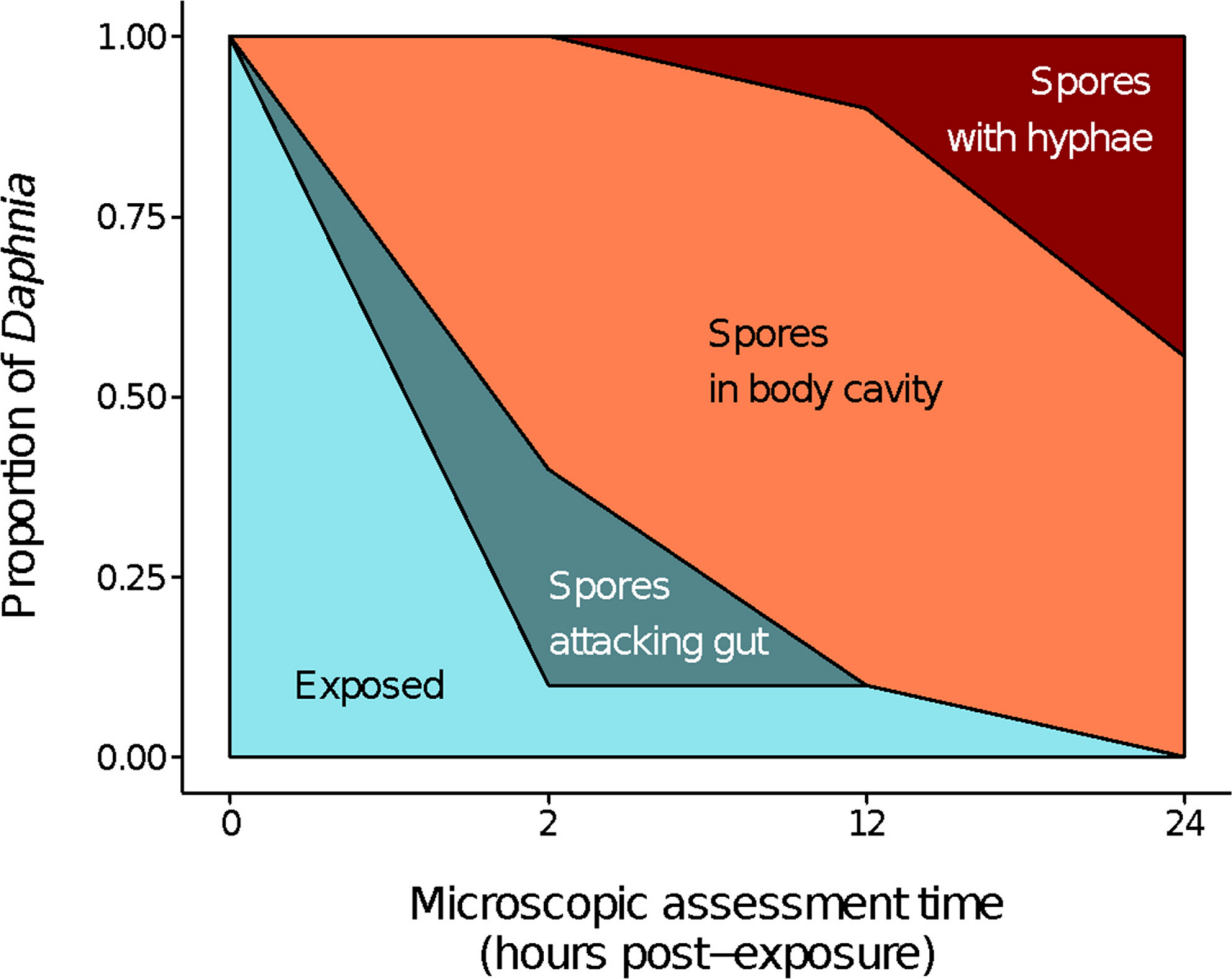
The first 24 h of exposure are an opportune time to investigate changes in gene expression because it is a critical period for host immune defenses. This graph displays the proportion of *Daphnia* on the y‐axis that is at a specific stage of infection (shaded light blue for exposed, sage green for “spores attacking gut”, orange for “spores in body cavity”, and red for “spores with hyphae”) over time (hours) on the x‐axis. At time 0, all individuals are exposed and by 24 h all individuals have spores in the body cavity (with or without hyphae).

The additional *Daphnia* collected per exposed treatment were examined microscopically to evaluate the stages of infection at 2 h (*N* = 10), 12 h (*N* = 10), and 24 h post‐exposure (*N* = 9) to observe what was occurring inside the host as a reference point for understanding the RNA sequencing results. These microscopic evaluations can be damaging and, in some cases, can lead to the death of the observed host. We, therefore, chose to include additional *Daphnia* for microscopic observation (rather than observing individuals sampled for RNA‐seq) to ensure that whole, intact *Daphnia* were used for RNA extraction, thereby minimizing variation in our RNA‐seq data. Pathogen‐exposed *Daphnia* were examined using compound microscopy (400× magnification) following Stewart Merrill and Cáceres (2018). In brief, the full body of each individual was visually scanned and particular attention was paid to the gut epithelium, where spores must cross into the body cavity to infect the host. We characterized *Daphnia* with four stages (Figure 2). “Exposed” denotes that the *Daphnia* had consumed infective spores (which could be observed inside the lumen of the gut), but the spores had not yet made contact with the gut epithelium. “Attacked by spores” denotes that at least one infective spore had pierced the gut epithelium but had not fully crossed into the body cavity. “Spores in body cavity” denotes that at least one spore had fully crossed the gut epithelium and entered the body cavity. Finally, “Spores with hyphae” denotes that at least one spore in the body cavity had germinated, producing fungal hyphae. Individuals were classified based on the most advanced stage present.

### 2.2 Transcriptome assembly and differential gene expression

To measure differences in gene expression, we homogenized each replicate of *Daphnia* using a TissueLyser II (Qiagen, Valencia, CA). We extracted total RNA from each replicate using the Qiagen RNeasy kit following the manufacturer's instructions. We assessed RNA concentration via a nanodrop, with acceptable concentrations lying between 3.5 ng/μL and 133 ng/μL. Additionally, RNA quality and quantity were assessed by the Carver Biotechnology Center at the University of Illinois Urbana‐Champaign using a Bioanalyzer and Qubit (RIN scores for libraries ranged from 6.4 to 8.4). We prepared cDNA libraries for sequencing using Illumina's “TruSeq Stranded mRNA‐seq Sample Prep kit”. The 18 libraries were pooled and sequenced on two lanes (nine pools/lane) on an Illumina HiSeq 4000 (paired‐end, 2 × 100 nt). Raw fastq files were generated and demultiplexed with *bcl2fastq* (v2.17.1.14) conversion software.

Reads were assembled into a transcriptome by HPCBio (High‐Performance Computing in Biology) at the University of Illinois Urbana‐Champaign. We trimmed adapters and low‐quality bases from reads using Trimmomatic (v0.38) with parameters ILLUMINACLIP: TruSeq3‐PE‐2.fa:2:15:10 LEADING:28 TRAILING:28 MINLEN:30. Next, we removed *M. bicuspidata* sequences from the reads by alignment to both *M. bicuspidata* genome assemblies (accession numbers: GCF_001664035.1, GCA_003614695.1) available in NCBI Genbank database. We assembled the remaining reads de novo using Trinity v. 2.10.0 (Grabherr et al., 2011) to both individual transcripts and groups of similar transcripts comprising a “gene.” To measure transcriptome completeness, we used BUSCO v. 3.0.1 (Manni et al., 2021; Simão et al., 2015) to scan for the presence of genes highly conserved across the arthropods.

We quasi‐mapped sequenced reads to the de novo transcriptome using Salmon v. 1.0.0 (Patro et al., 2017) via Trinity's (v. 2.10.0) wrapper script align_and_estimate_abundance.pl. We summed individual transcript counts to gene level while adjusting for transcript length differences to provide more accurate gene‐level quantification (Soneson et al., 2015). To quantitatively measure the degree of differential gene expression among samples, we ran gene counts through standard filtration with a threshold of 0.5 counts per million in at least three samples. Due to the amount of variation (the number of fragments/library ranged from 18 to 92 million, Table S1), we performed a limma‐voom normalization (Law et al., 2014) and used normalized values in a two‐way ANOVA model using the limma package (version 3.44.3; Ritchie et al., 2015) to test for main effects of time, treatment, and their interaction (*R* version 4.0.4). Additionally, to account for between‐sample variation, we used the remove unwanted variation (RUVSeq) normalization method to detect covariance structures present in the data unrelated to time or treatment (as a result of batch effects, library preparation, or other technical effects) (Risso et al., 2014). This analysis yielded four co‐variates which we added to the statistical model to adjust for their effects. The final model included treatment (exposed vs. control), time (2, 12, and 24 h), and the four covariates. Following this, we made nine pairwise comparisons between all treatment and time groups, as well as testing whether the effect of time at 12 and 24 h depended on the treatment group and performed an FDR adjustment globally to correct for multiple pairwise comparisons. Since this is an exploratory analysis to identify novel immune genes for future work, we considered transcripts differentially expressed at an FDR‐adjusted *p*‐value of <.1 for further analysis.

### 2.3 Annotation and GO enrichment analysis

The de novo transcriptome was annotated using Trinotate v. 3.2.1 (Bryant et al., 2017) to compare assembled transcripts to publicly available data (SwissProt, release 2020_06), protein domain identification (HMMER v. 3.2.1, Pfam v. 33.0), protein signal peptide and transmembrane domain prediction (signalP v. 4.1, tmHMM v. 2.0c), open reading frames (TransDecoder v. 5.5.0), and ribosomal RNA (RNAmmer v. 1.2). Functional annotations of each transcript were called from Gene Ontology (GO) and Kyoto Encyclopedia of Genes and Genomes (KEGG). To assess the biological significance and identify which functional categories were enriched among the differentially expressed transcripts, a GO term enrichment analysis including Biological Processes (BP) was performed using the *topGO* package in *R* version 4.0.4 (Alexa & Rahnenführer, 2020). A Kolmogorov–Smirnov test was used to determine the enrichment significance for each GO term, and those with a *p*‐value < .001 were taken as significant.

## 3 RESULTS

### 3.1 Observations of the host‐pathogen interaction

The proportion of pathogen‐exposed *Daphnia* in each stage of infection over the first 24‐h time period is provided in Figure 2. At 0 h post‐exposure (the time point of experimental inoculation) all *Daphnia* in the experimental treatment were exposed to fungal spores by virtue of the experimental design. By 2 h post‐exposure, we observed that 90% of *Daphnia* had moved beyond the “exposed” stage: 30% were attacked by spores, and 60% had spores that had successfully entered the body cavity. By 12 h post‐exposure, 80% of *Daphnia* had spores inside the body cavity, and 10% possessed germinating hyphae (the remaining 10% were still in the “exposed” stage). By 24 h post‐exposure, all *Daphnia* (100%) were infected with spores in the body cavity: 55% were still at the spore stage, and 44% possessed germinating hyphae (Figure 2). Based on the timing of this interaction, we might expect that gene expression changes related to the attack of the gut epithelium occur at 2 h, changes related to invasion of the body cavity occur at 12 h, and changes related to the progression of infection (hyphae) occur at 24 h.

### 3.2 Transcriptome assembly and annotation

The de novo trinity assembly resulted in 208,350 transcripts from 99,609 genes (full assembly statistics shown in Table S2). The BUSCO results of the de novo transcriptome included 99.4% of genes conserved across the phylum Arthropoda, suggesting completeness of the assembly. Moreover, at least 93% of reads mapped back to the transcriptome (Figure S1). After summing from transcript to gene level, 22,637 genes (23%) met standard filtration thresholds (0.5 counts per million in at least three samples) which is typical for a transcriptome assembly that includes excess transcripts/genes due to sequencing errors (Mbandi et al., 2014). Of the retained genes, 53% (12,067 of 22,637) were successfully annotated to a functional class using six different Gene Ontology databases from Trinotate. All 22,637 genes as well as their annotations, average expression, fold change, raw and FDR‐adjusted *p*‐values, are shown in Table S3.

### 3.3 Differential gene expression and GO enrichment analysis

**FIGURE 3:**
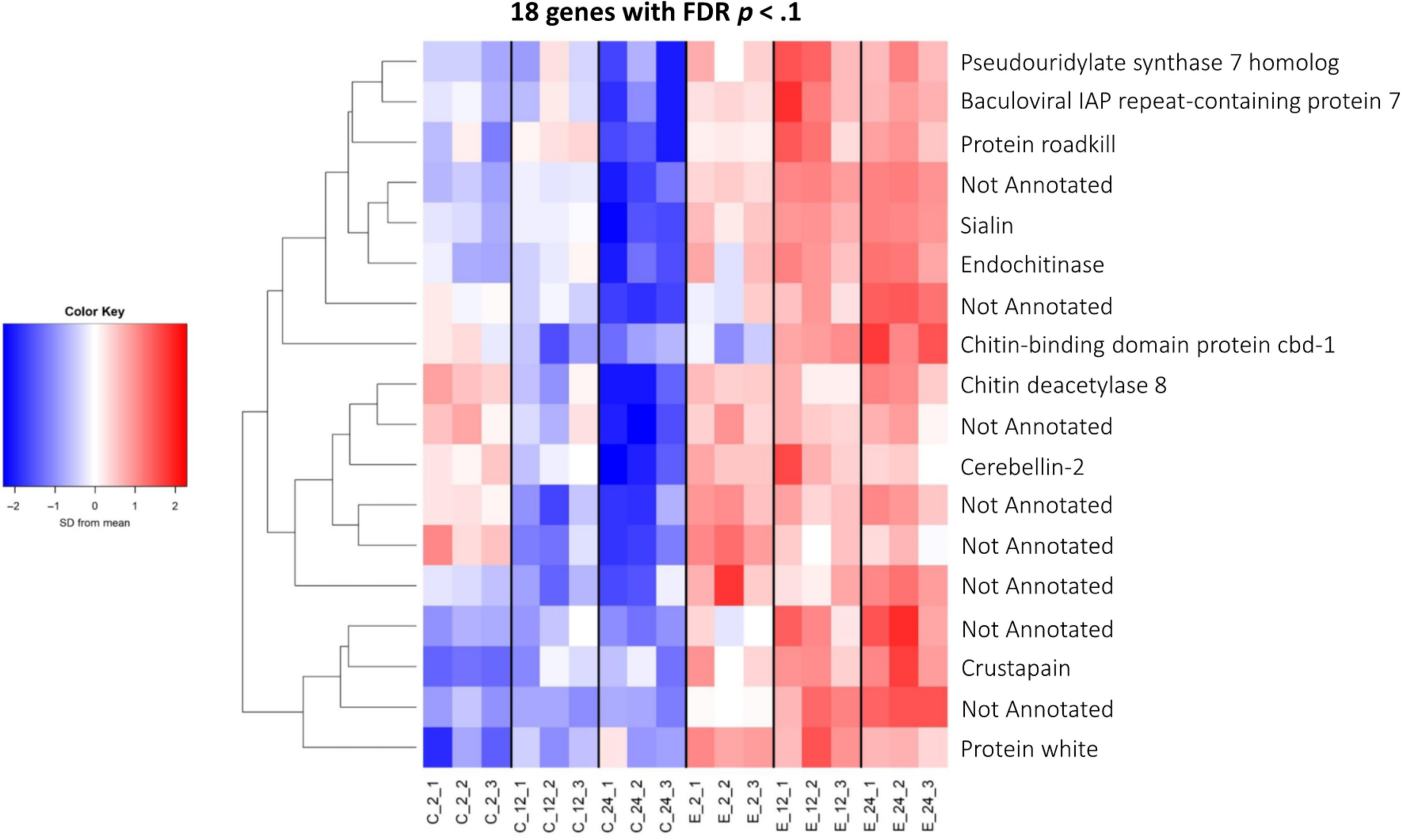
There are more genes that are differentially expressed as a function of time than exposure to the pathogen, and all DEGs from exposure are upregulated in exposed groups compared to control (unexposed) groups. The 18 genes differentially expressed as a result of exposure to the fungal pathogen (FDR adjusted *p* < .1).

When we assessed how pathogen exposure influenced transcript expression, results of the two‐way ANOVA showed only 18 genes with significant main effects (FDR *p*‐value < .1, averaged over all time periods), all of which were upregulated in exposed groups (Figure 3; two‐way ANOVA F statistics, raw, and adjusted *p*‐values shown in Table S4). Among these, 10 were annotated from GO databases. The gene ID, BLASTX symbol and name, fold change, raw *p*‐value, FDR‐adjusted *p*‐value, and GO terms for the differentially expressed (DE) genes from pathogen exposure and time are shown in Tables S4 and S5, respectively. The overall effect of time was more nuanced, with 12 and 24 h showing more up‐regulated transcripts than both groups at 2 h, but more variation between replicates. When comparing the main effects of time, there were 2754 genes that were differentially expressed at an FDR of *p* < .1 (Figure S2).

**FIGURE 4:**
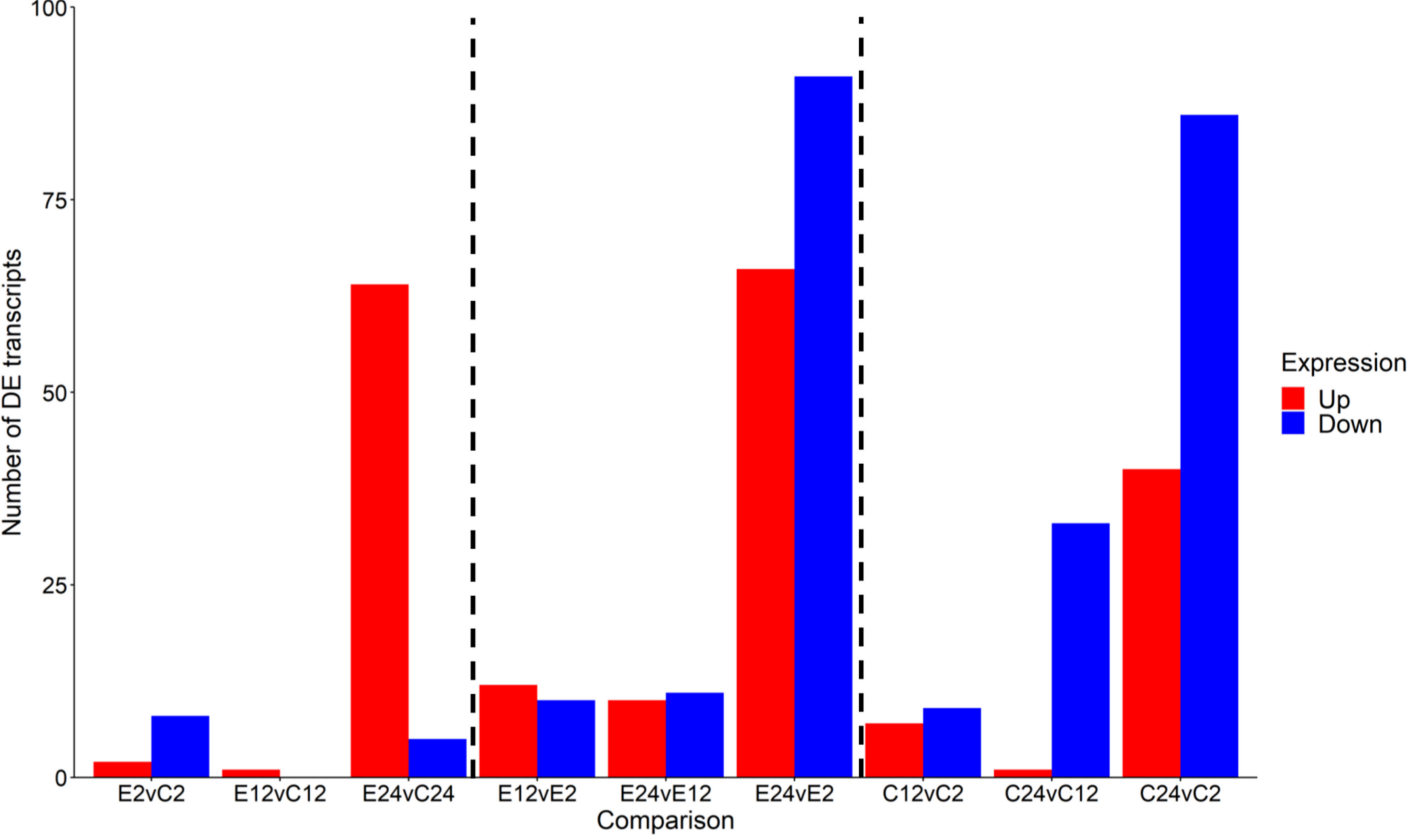
The total number of up and downregulated transcripts for each pairwise comparison where “E” abbreviates “Exposed,” “C” abbreviates “Control,” and 2, 12, and 24 represent the hours that had passed since the initial treatment and the time point at which the sample was collected. Black vertical dashed lines separate the three main group comparisons (exposed vs. control), exposed groups at the three time points, and control groups at the three time points. These results visualize the rapid and transient regulatory response to pathgoen exposure in *D. dentifera*.

When we assessed specific pairwise comparisons between treatment and time, no genes were shared among all pairwise comparisons. When comparing exposed to unexposed groups at 24 h (E24vC24), there are more DE genes that are upregulated, however, when comparing within groups at 24 h (E24vE2 and C24vC2) there are more downregulated genes (Figure 4). When we compared changes over time between treatment groups, we found changes in the number of differentially expressed genes from 10 when comparing E2v C2, down to only one DE gene when comparing E12vC12, which rapidly increases to 69 when comparing E24vC24 (80 genes total). Of these, five genes were shared between at least two of the three time‐point comparison groups, but no genes were shared between all three time‐point comparison groups (E2vC2, E12vC12, and E24vC24) suggesting that it is not necessarily the same genes that are being up‐ or downregulated over time. However, there was limited statistical power due to the low sample size.

**FIGURE 5:**
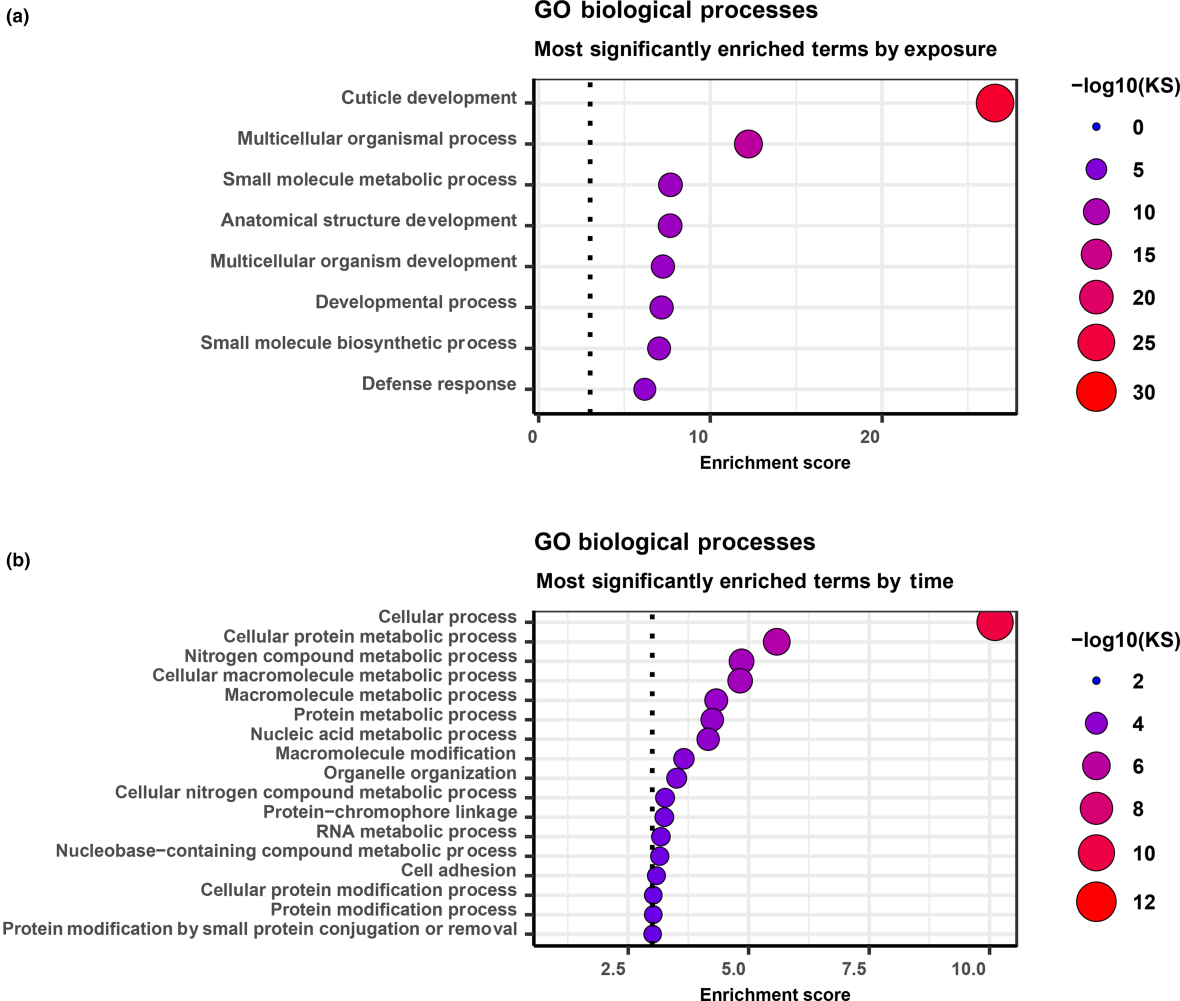
The DE transcripts upregulated as a function of pathogen exposure are associated with GO terms linked to immune function. (a) A GO enrichment analysis depicting the most enriched GO terms (Biological Processes) from transcripts differentially expressed due to pathogen exposure (FDR adjusted *p* < .1). (b) The most enriched GO terms from transcripts differentially expressed as a function of time at an FDR‐adjusted *p* < .1. The x‐axis indicates the enrichment score, and the y‐axis shows each respective GO term. Point color and size indicate the significance of log K‐S *p*‐values. The horizontal dotted line indicates a K‐S *p*‐value < .001.

Two GO enrichment analyses were performed, one with the 18 differentially expressed genes due to pathogen exposure, and one with the 2754 differentially expressed genes as a main effect of time. The most significantly enriched GO terms (Biological Processes) for each set of differentially expressed genes are shown in Figure 5a for pathogen exposure and Figure 5b for time (K‐S significance test with *p* < .001). While there were far more DE transcripts as a function of time, many of those that were significantly upregulated due to pathogen exposure were of immunological interest, as shown by the GO enrichment analysis. Examples of those that were highly enriched as a function of pathogen exposure were cuticle development (*p* < .0001) and defense response (*p* < .0001) (Figure 5a). All terms, along with their annotations and associated p‐values are shown in Table S6 for time and Table S7 for pathogen exposure.

## 4 DISCUSSION

Invertebrate immunity is driven by diverse, complex, and likely adaptive mechanisms; however, the genomic basis of immunity remains largely unexplored across invertebrates (Kurtz & Franz, 2003). Genomic studies provide a basis for an enhanced understanding of the diversity of physiological, behavioral, and evolutionary components underpinning defenses against pathogens (Hajek & Shapiro‐Ilan, 2018; Hudson, 2008). More specifically, RNA‐seq techniques can deliver novel insight into dynamic changes happening at the gene expression level in response to pathogen exposure, particularly for organisms for which there is no sequenced genome (Huang et al., 2016). Here, we used RNA‐seq analyses to investigate gene expression responses in *D. dentifera* to the common fungal pathogen, *M. bicuspidata*, in the first 24 h following pathogen exposure. Our findings (1) indicate that *Daphnia* quickly mount a dynamic immune response following pathogen exposure, (2) provide a group of immunologically relevant genes from which future work in this area can be pursued, and (3) suggest that cuticle development plays an important role in *Daphnia* immunity.

Determining the genetic basis of immunity is an essential step in advancing the field of eco‐immunology (Agrawal et al., 2007; Brock et al., 2014; Cáceres et al., 2014; Schoenle et al., 2018). The realization that much is unknown about the intricacy of invertebrate immunity is a common denominator among current genomic investigations (Cáceres et al., 2014; Decaestecker et al., 2011; Hall & Ebert, 2012). A study involving infected and uninfected *Daphnia magna* used qRT‐PCR to examine changes in expression levels of five genes that were shown to be immune‐related in other organisms (Decaestecker et al., 2011). However, only weak patterns of gene expression were detected. Likely, the target genes referenced from other organisms were not representative of *Daphnia* immune‐related genes and thus limited the detection of dynamic changes in gene expression levels. Our study presents a substantial advance in that we have assembled the transcriptome de novo, rather than focusing on target genes, to take a holistic look at which genes are being differentially expressed in response to pathogen exposure.

As the amount of time, since inoculation increased, the number of DE genes between the exposed and unexposed treatment groups increased. This aligns with the progression of infection in *Daphnia* when exposed to *Metschnikowia* (Figure 2). At 2 h post‐exposure, some hosts have ingested spores that have not yet punctured the gut whereas other hosts already have spores present in the body cavity. By 24 h, 100% of *Daphnia* have spores present in their body cavity, some of which have already progressed to the hyphal stage. Figure 4 visualizes the number and direction of DE genes at each time‐point comparison, emphasizing the rapid and dynamic transcriptional response to pathogen exposure in *D. dentifera*. In all treatment groups, 24 h shows the largest difference in the number of differentially expressed genes, suggesting that this may be a pivotal point in development as well as immune response. Similar time‐sensitive responses have been seen in other systems. For example, a cellular response in which hemocytes are released to combat infection has been observed in *D. magna* just hours following exposure (Auld et al., 2010). Our study was designed to capture the immunological events occurring in this early time period, which are critical for determining whether the host will combat or succumb to infection (Stewart Merrill, Hall, & Cáceres, 2021; Stewart Merrill, Rapti, & Cáceres, 2021). Beyond this early window, if they are not cleared by the initial immune response, spores develop into a series of morphological stages, ultimately killing the host 9–10 days after pathogen inoculation (Stewart Merrill & Cáceres, 2018). In the future, similar studies extending beyond the first 24 h of exposure will provide a more holistic perspective of the genetic interplay of host and pathogen. It is also important to note that circadian rhythm has been shown to drive infection risk in the *D. dentifera‐M. bicuspidata* system, and our results could be shaped by this relationship (Pfenning‐Butterworth et al., 2022).

Decaestecker et al. (2011) failed to find differential gene expression in response to infection when specifically targeting genes coding for prophenoloxidase, two nitric oxide synthases, alpha‐2‐macroglobulin, and arginase, all of which have been established as core components of invertebrate immune systems (McTaggart et al., 2009). We leveraged transcriptomic analysis to provide unbiased insight into the processes happening during infection. Interestingly, when we look at our annotated genes, multiple GO terms involved with immunity were detected among the most significant GO terms in our dataset. However, the immune‐related genes found in *D. magna* were not differentially expressed in our sample of *D. dentifera*, further emphasizing the potential diversity of immune responses among invertebrates. Of particular interest, the first and second most significant GO terms in our dataset were related to cuticle development. Through microscopic visualization, we found that a spore passing the gut epithelium is a critical step for the success of the spore in the first 24 h following exposure. Prior work has established that the penetrability of the gut epithelium is a key factor underlying variation in resistance among *Daphnia* genotypes and individuals (Stewart Merrill et al., 2019; Stewart Merrill, Hall, & Cáceres, 2021; Stewart Merrill, Rapti, & Cáceres, 2021). In our study, we predicted that such variation might be present in this physical host response and this prediction was supported by the dramatic upregulation of cuticle‐related genes we have found. Cuticular melanin is involved in an insect encapsulation immune response in which host hemocytes form a melanizing capsule around a pathogen to inhibit its spread (Fedorka et al., 2013; Nappi & Christensen, 2005). The cuticle itself is the location in which vital immunological molecules, such as melanin or Pro‐phenoloxidase, are transported and regulated (Asano & Ashida, 2001; Cerenius & Söderhäll, 2004). Notably, these findings conflict with previous RNAseq studies in which proteins related to chitin, a molecule that strengthens the cuticle, were found to be downregulated in response to pathogen exposure in both *D. galeata* and *D. magna* (Lu et al., 2018; McTaggart et al., 2015). The difference in regulatory response could be due to differences in pathogen infection strategies (i.e., *Metschnikowia* punctures the gut epithelium while *Pasteuria* and *Caullerya* do not). Nonetheless, our findings strongly emphasize the importance of the cuticle and its development in the invertebrate immune response. Perhaps the candidate genes in our study and those of McTaggart et al. (2015) and Lu et al. (2018) provide a link to deepening our understanding of gut permeability and its relationship to immunity.

Of the other most significant GO terms were immunologically relevant genes such as prostaglandin metabolic processes and hydrogen peroxide metabolic processes (Table S7). Prostaglandin is a lipid mediator that plays a role in the regulation of the innate immune system in insects and both innate and acquired immune processes in vertebrates through its effects on immune cells such as macrophages and T‐cells (Ahmed et al., 2018; Martínez‐Colón & Moore, 2018). Hydrogen peroxide, which was upregulated in this study at 2 and 24 h, also plays a role in immunity as a reactive oxygen intermediate (ROI). ROIs have been shown to mediate cell proliferation and apoptosis, are potentially toxic to pathogens and activate a transcription factor involved in the immune response of vertebrates (Nappi & Vass, 1998). Notably, hydrogen peroxide is one of the main players in mounting an inflammatory response along with initiating many other signaling cascades (Wittman et al., 2012). In addition to immune‐related functions, developmental and metabolic functions such as anatomical structure development, hormone processes, and glucose metabolic processes, were highly upregulated among our treatment groups. Anatomical structure development could be related to wound healing, which makes sense given the puncture infection strategy of *M. bicuspidata*. Upregulation of metabolic and hormonal processes suggests that there could be a potential relationship between metabolism and hormonal function and the immune response that is not fully understood. It is interesting to note, however, that Lu et al. (2018) found suppression of general metabolic function in *D. galeata* exposed to an ichthyosporean parasite whereas we found upregulation of metabolic genes. This could be due to the difference in time at which individuals were sacrificed (at 48 h for Lu et al. (2018) vs. our earlier time points of 2, 12, and 24 h) and/or the level of specificity of the host–parasite interaction which could affect the resulting expression profile. Given that these studies were conducted in two distantly related species of *Daphnia* in response to pathogens belonging to two different families, it is not overly surprising that the transcriptional response to infection differed between the two species and suggests that diverse defense mechanisms are present in *Daphnia*.

Intriguingly, many of our most significant GO terms lacked a clear relation to immunity. This is not to say that these GO terms are not immunologically relevant, but that a possible relationship may not yet be understood. Moreover, 46% (10,570 of 22,637) of the genes identified in this study lacked any GO annotation, and 45% of all differentially expressed genes lacked any GO annotation (1248 or 2772). Revealing the functions of these genes and investigating the physiological cascade initiated by the differential gene expression we observed, will likely lead to the discovery of novel immune mechanisms (Long, 2001; Singh & Ahi, 2022).

Using RNA‐seq, we were able to produce the first gene expression analysis of *D. dentifera* without the use of target genes, enabling a comprehensive investigation of putative immune‐related genes. Of the most significant GO terms in exposed *Daphnia*, were those associated with cuticle development, defense responses, prostaglandin, and hydrogen peroxide activity. These terms and molecules have known and, in some cases relatively unresolved, immunological importance that call for further exploration. Particularly, our findings suggest that investigating the role of the cuticle in immunity to pathogens is a strong avenue for future studies. Other significantly upregulated genes which lacked annotation are also likely to be related to the invertebrate immune response and provide exciting prospects for further analysis. Studies in which these candidate genes are knocked out (e.g., Liu et al., 2016; Wang et al., 2010; Zhang et al., 2017) could lead to an enhanced understanding of the genetic basis for immunity in *Daphnia*, or the discovery of novel immune mechanisms. Further, extending the outlook on gene expression in response to infection beyond the first 24 h would likely lead to the discovery of more putative immune‐related genes, since infection extends days beyond our observed time period. With a suite of immunologically relevant genes, we have laid a foundation from which further investigations of immune function in *Daphnia* can be pursued. In utilizing this model system, we can glean key insights into the bigger picture of invertebrate immunology.

## AUTHOR CONTRIBUTIONS

**Emily E. Terrill Sondag:** Formal analysis (equal); investigation (equal); visualization (equal); writing – original draft (lead). **Tara E. Stewart Merrill:** Conceptualization (lead); data curation (lead); funding acquisition (equal); methodology (equal); writing – review and editing (equal). **Jenny Drnevich:** Data curation (equal); formal analysis (equal); writing – review and editing (equal). **Jessica R. Holmes:** Data curation (equal); formal analysis (equal); writing – review and editing (supporting). **Eva K. Fischer:** Formal analysis (equal); writing – review and editing (equal). **Carla E. Caceres:** Conceptualization (equal); formal analysis (equal); funding acquisition (equal); methodology (equal); resources (equal); supervision (equal); writing – review and editing (equal). **Lynette R. Strickland:** Formal analysis (equal); investigation (equal); methodology (equal); supervision (equal); visualization (lead); writing – review and editing (equal).

## Supporting information


Figure S1.

Figure S2.

Table S1.

Table S2.

Table S3.

Table S4.

Table S5.

Table S6.

Table S7.

## Data Availability

Raw sequencing data as well as pooled library metadata from which these analyses are based are available on Dryad at: Terrill, Emily et al. (2023), RNA sequencing for individuals of *Daphnia dentifera* exposed and unexposed to a fungal pathogen, Dryad, Dataset, https://doi.org/10.5061/dryad.r7sqv9sfj.
