# Supplementary figures and images for: Differential gene expression in response to fungal pathogen exposure in the aquatic invertebrate, Daphnia dentifera
Source: Ecol Evol. 2023 Jul 28;13(8):e10354. doi: 10.1002/ece3.10354 (PMC10375369; doi:10.1002/ece3.10354)

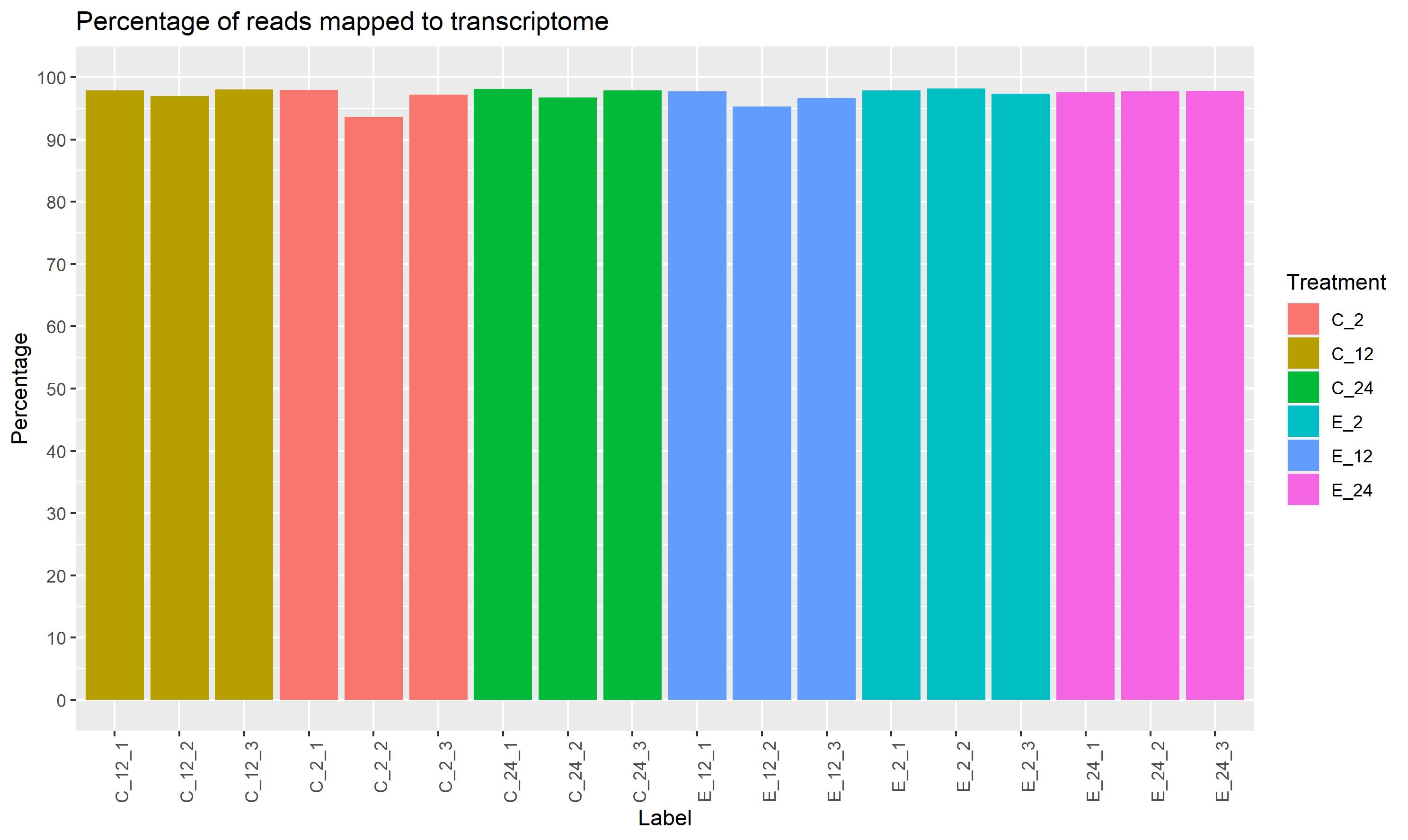

Supplement: Supplementary file 1 — Figure S1. [file ECE3-13-e10354-s001.jpeg]

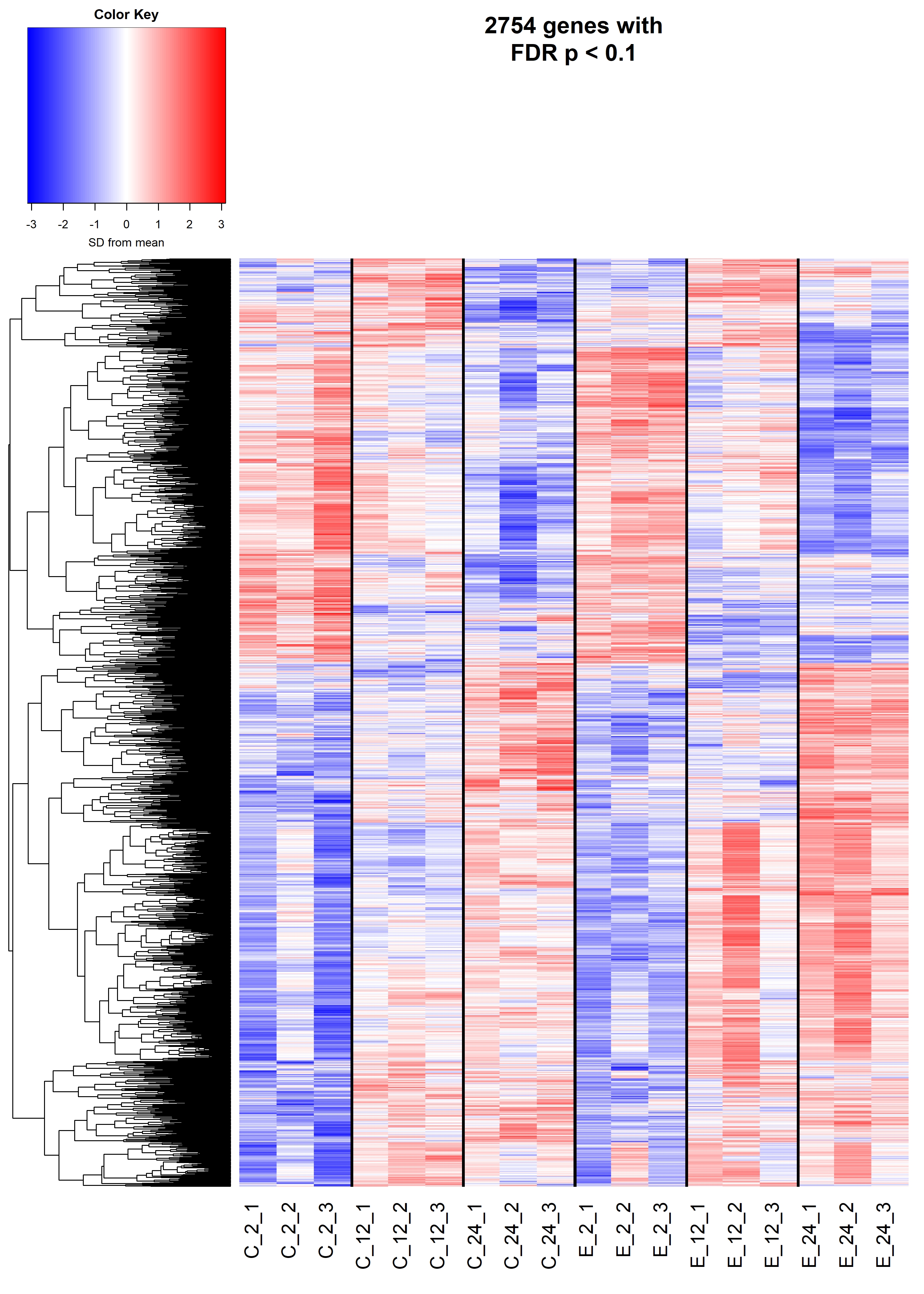

Supplement: Supplementary file 2 — Figure S2. [file ECE3-13-e10354-s003.jpeg]
